# WeederH: an algorithm for finding conserved regulatory motifs and regions in homologous sequences

**DOI:** 10.1186/1471-2105-8-46

**Published:** 2007-02-07

**Authors:** Giulio Pavesi, Federico Zambelli, Graziano Pesole

**Affiliations:** 1 Dipartimento di Scienze Biomolecolari e Biotecnologie, University of Milan, Milan, Italy; 2 Dipartimento di Biochimica e Biologia Molecolare "E. Quagliariello", University of Bari, Bari, Italy; 3 Istituto Tecnologie Biomediche – Consiglio Nazionale delle Ricerche, Bari, Italy

## Abstract

**Background:**

This work addresses the problem of detecting conserved transcription factor binding sites and in general regulatory regions through the analysis of sequences from homologous genes, an approach that is becoming more and more widely used given the ever increasing amount of genomic data available.

**Results:**

We present an algorithm that identifies conserved transcription factor binding sites in a given sequence by comparing it to one or more homologs, adapting a framework we previously introduced for the discovery of sites in sequences from co-regulated genes. Differently from the most commonly used methods, the approach we present does not need or compute an alignment of the sequences investigated, nor resorts to descriptors of the binding specificity of known transcription factors. The main novel idea we introduce is a relative measure of conservation, assuming that true functional elements should present a higher level of conservation with respect to the rest of the sequence surrounding them. We present tests where we applied the algorithm to the identification of conserved annotated sites in homologous promoters, as well as in distal regions like enhancers.

**Conclusion:**

Results of the tests show how the algorithm can provide fast and reliable predictions of conserved transcription factor binding sites regulating the transcription of a gene, with better performances than other available methods for the same task. We also show examples on how the algorithm can be successfully employed when promoter annotations of the genes investigated are missing, or when regulatory sites and regions are located far away from the genes.

## Background

Genome sequencing projects have told researchers *where *genes are located, in human and an ever increasing number of other species, and microarrays and other sources of information can tell *when *genes are activated: but the complete understanding of *how *genes expression is regulated at the transcriptional and post-transcriptional levels, as well as the characterization of all the elements involved in the process still remain an open question in molecular biology. Transcription is a fundamental step in the regulation of gene expression, and it is modulated by the interaction of transcription factors (TFs) with their corresponding binding sites (TFBS) on the DNA [1], mostly located near the transcription start site (TSS) of the gene or far apart organized in cis-regulatory modules (CRMs, i.e. enhancers, silencers, etc.).

Computational methods for the discovery of conserved TFBSs (*motifs*) can be split into two broad categories: the 'single species, many genes' approach [2], and the 'single gene, many species' one [3]. In the former case, a set of regions (i.e., promoters) from co-regulated genes are analyzed looking for over-represented motifs, that is, the TFBSs responsible for the co-regulation of the genes; while in the latter approach, known as *phylogenetic footprinting *(a term first introduced in [4]), a single gene is investigated, and non coding regions flanking it are compared to their homologs in other species. Non coding sequence elements that are found to be conserved by evolution are likely to be involved in the regulation of the expression of the gene. Clearly, the two approaches can be merged, and each of a set of co-regulated genes can be compared both to its homologs, and to the others [5,6], and this analysis can be also performed on a full-genomic scale [7-10]. Given the ever increasing number of annotated genomic sequences available, phylogenetic footprinting has become more and more widely used, since it avoids the need of assembling a set of co-regulated genes (that in turn implies the need of building reliable datasets) and allows for the investigation of single genes alone.

The comparison of homologous non coding sequences can be performed both for the identification of single TFBSs, for example in promoter regions, and on a larger scale for the discovery of distal CRMs, an approach that has been successful in several cases ever since the first human-mouse comparisons have been possible (see [3] for a review). The available methods that are more commonly used first build an alignment (either local or global) of the sequences investigated [11], or take advantage of the pre-computed full genomic alignments now available [12,13]. Then, one simple solution to identify conserved functional elements is to use descriptors of the binding specificity of TFBSs (like *position specific weight matrices *[14] provided for example in the TRANSFAC database [15]) and look for conserved aligned regions fitting the descriptor. This approach can be used both for the detection of single TFBSs (see for example [16]) as well as of clusters of TFBSs likely to form conserved CRMs (among many others, [17,18]).

Methods of this kind have to face two issues: first of all, the need of reliable descriptors of the binding specificity of the different TFs. Usually, PWMs yield a large number of false positive matches [19], and while requiring a match to be conserved throughout different sequences reduces them, the problem of defining whether a match is significant in all the species considered remains. Second, and most important, is the need of having a reliable alignment of the sequences investigated. TFBSs tend to be quite short (6–15 nucleotides), when compared to a typical region analyzed (a promoter of 500–1000 bps): in case the sequences aligned are too divergent, the result is that conserved TFBSs can be missed simply because not correctly aligned. A dual solution, of using matches of TFBSs as anchors for the alignment has indeed been proposed in order to overcome this problem and improve alignment reliability [20]. Sequence alignments are avoided by the Footer algorithm [21], that performs human-mouse comparisons by using distinct descriptors for the TFBSs of the two species, and looks for matches for homologous TFs that fall at similar positions with respect to the genes studied.

When known TFBSs descriptors are not used, the idea is naturally to identify elements or regions that can be considered to be "significantly conserved", and hence likely to possess a regulatory function. The simplest strategy is just to single out the most conserved parts of the alignments, according to identity percentage: while a non-coding region highly (or "ultra") conserved can be reasonably suspected to possess a functional role [22,23], the problem is often to define how much conserved regions should be to be considered significant. In fact, while conserved TFBSs and CRMs can be qualitatively defined as "islands of conservation in a sea of much less conserved DNA" [24], suitable measures able to quantify this concept have to be introduced [25]. Indeed, recent research has focused on this point. MBA is an algorithm that looks for blocks of highly constrained alignments, weights them according to phylogenetic distance, and estimates significance according to neutral substitution rates [26]. A regulatory potential (RP) score is defined in [27], by looking for patterns of conservation frequently found in conserved regulatory regions. Evolutionary and hidden Markov models are combined in phastCons [28], in order to define a measure of significance for the conservation of a multiple genomic alignment.

While all these methods can perform well in the identification of quite large conserved regulatory regions like CRMs or whole promoters [29], they are less powerful for the identification of single TFBSs. Size and conservation of TFBSs are in fact often not enough to constitute "significant" parts of the alignments (or significant local alignments) [30]. Another important issue is defining how much conserved a region should be to be considered as worth of further investigation. Different homologous genomic regions, for example in a human-rodent comparison, show a varying degree of conservation, that is, seem to evolve at different rates [31]. Thus, for example, if a unique significance threshold is used in some cases a whole promoter region can be considered as "significantly conserved" (thus missing the locations of single TFBSs), while in others no significant sequence element is reported.

Indeed, some methods that do not compute a global or local sequence alignment have already been proposed. Several motif discovery algorithms for the detection of conserved sequence elements in co-regulated genes already exist [2,32], and sometimes have been successfully applied to homologous sequences as well [33]. Algorithms of this kind, however, assume that input sequences are not related by evolution, and thus look for subtle similarities: the result is that a human-mouse comparison can report a deluge of conserved motifs, regardless of the algorithmic strategy and significance evaluation employed.

Footprinter [34], instead, is an algorithm for the discovery of conserved motifs explicitly devised for phylogenetic footprinting, that looks for conserved sequence motifs making use of the phylogenetic relationships among the sequences. Motifs conservation is first of all evaluated according to parsimony scores. While this approach bypasses the need of pre-aligning the sequences, parsimony scores alone do not provide a fine-grained ranking of the motifs found, especially when a few sequences are investigated, i.e., in a typical human-rodent comparison. A statistical evaluation of the results of the algorithm is possible, by comparing motifs found with a "random" dataset of simulated orthologous sequences. The problem is, again, that even when the same species are compared the degree of conservation in the promoters of different genes can vary significantly according to the genes investigated, and thus establishing unique significance thresholds has the effect of yielding too many significant motifs in some cases, too few in others.

The aim of this work is to introduce a novel strategy to identify significantly conserved motifs and regions in homologous sequences. Given a reference sequence, and one or more homologs, the algorithm we propose is based on the idea that functional conserved elements should be conserved both in sequence and in position with respect to the genes they regulate. Starting from this consideration, we adapted to this case a statistical measure we previously used for the discovery of TFBSs in sequences from co-regulated genes, by adding to it positional conservation. Moreover, as we discussed before, defining absolute measures of significance for conservation is hard, given that sequence conservation varies greatly according to the species and the genes considered. We tackle this problem by measuring conservation not in an absolute, but in a relative way, according to the average degree of conservation of the whole sequences compared, with the idea that functional elements should be more conserved than the rest: in other words, what the algorithm evaluates is not significant conservation, but rather significant variation of conservation within the same sequences. Clearly, the motifs and regions selected by the algorithm can be further processed by matching them against descriptors of known TFBSs, or compared to regions extracted from other co-regulated genes.

## Results

In this section we first present the algorithm, then we assess its performance showing results obtained on tests performed on collections of known functional elements.

### Algorithm

The WeederH algorithm takes as input a reference sequence *S*, and any number *k *≥ 1 of homologous sequences *H*_1 _... *H*_*k*_. Also, it assumes that all the sequences have been taken from the different genomes with respect to the same reference points: that is, all sequences are upstream of the TSS or the ATG codon of homologous genes, or are intergenic regions between two genes and between their homologs in other species, and so on. Conserved motifs are identified in the reference sequence, by comparing it to the homologs. The steps performed by the algorithm can be summarized as follows:

1. Each oligo of suitable size of the reference sequence is matched against the homologous sequences;

2. Matches not exceeding a given substitution threshold are scored with a measure taking into account sequence and position conservation, and the highest scoring match is kept;

3. Oligo scores are transformed into relative scores, according to the average scores obtained by oligos of the same size;

4. High scoring oligos are merged, whenever possible, in order to obtain longer motifs and regions.

It can be immediately seen that we used for modeling conserved sites the oligos themselves, rather than more involved representations of TFBSs like profiles and position weight matrices [14]. The latter are clearly more powerful than oligos and consensi for modeling the binding specificity of a given TF: however, for the ab initio discovery of novel motifs and sites, which in turn is essentially based on the detection of similar oligos and its statistical evaluation [2], recent results have shown no definite prevalence, and rather consensus- (or oligo-) based models have often yielded better results [32].

#### Finding conserved motifs

In the first step, each oligo of a given length *m *of the reference sequence *S *is matched against each of the homologous sequences, and all its occurrences with at most *e *substitutions are collected. Given *s*_*i*_, the *m*-mer at position *i *of the reference sequence, a match at position *j *of the *k*-th homologous sequence *H*_*k *_presenting *d *≤ *e *substitutions is scored by taking into account sequence and position conservation:

*B*_*k *_(*i*, *j*, *m*) = - log (*E *(*s*_*i*_, *d*, *k*)) - log (Δ (*i*, *j*) + 1)

Where *E(s*_*i*_*, d, k) *is the expected frequency of *s*_*i *_with at most *d *substitutions in the species of sequence *k *(see Methods), and *Δ (i,j) *is the distance between the two positions, measured according to the reference points defined (e.g. *i *and *j *bps upstream of the TSS of the respective genes). This function is similar to the one we employed in the original Weeder algorithm [35,36], that anyway did not make any assumption on the positional conservation of the motifs.

The score of oligo *s*_*i *_with respect to the *k*-th homolog *H*_*k *_is given by the maximum among the matching positions:

Bk(i,m)=max⁡jBk(i,j,m)
 MathType@MTEF@5@5@+=feaafiart1ev1aaatCvAUfKttLearuWrP9MDH5MBPbIqV92AaeXatLxBI9gBaebbnrfifHhDYfgasaacH8akY=wiFfYdH8Gipec8Eeeu0xXdbba9frFj0=OqFfea0dXdd9vqai=hGuQ8kuc9pgc9s8qqaq=dirpe0xb9q8qiLsFr0=vr0=vr0dc8meaabaqaciaacaGaaeqabaqabeGadaaakeaacqWGcbGqdaWgaaWcbaGaem4AaSgabeaakiabcIcaOiabdMgaPjabcYcaSiabd2gaTjabcMcaPiabg2da9maaxababaGagiyBa0MaeiyyaeMaeiiEaGhaleaacqWGQbGAaeqaaOGaemOqai0aaSbaaSqaaiabdUgaRbqabaGccqGGOaakcqWGPbqAcqGGSaalcqWGQbGAcqGGSaalcqWGTbqBcqGGPaqkaaa@4599@

If no match is found for *s*_*i *_in sequence *H*_*k*_, or all matches yield negative scores (that is, the distance exceeds the expected value) this score is set to zero. At this point, the overall score associated with *s*_*i *_could be defined as the sum of the scores in each homologous sequence: but, the *B*_*k*_*(i) *values can vary greatly according to the overall conservation of the sequences compared (e.g., a human-mouse comparison will yield scores greater than a human-chicken comparison). However, regardless of the species considered, the idea underlying the algorithm is that functional elements should be more conserved than the rest of the sequences: thus, instead of using directly the *B*_*k*_*(i) *scores the algorithm first transforms them into *relative *scores. Let

μ(k,m)=∑iBk(i,m)|S|−m+1
 MathType@MTEF@5@5@+=feaafiart1ev1aaatCvAUfKttLearuWrP9MDH5MBPbIqV92AaeXatLxBI9gBaebbnrfifHhDYfgasaacH8akY=wiFfYdH8Gipec8Eeeu0xXdbba9frFj0=OqFfea0dXdd9vqai=hGuQ8kuc9pgc9s8qqaq=dirpe0xb9q8qiLsFr0=vr0=vr0dc8meaabaqaciaacaGaaeqabaqabeGadaaakeaaiiGacqWF8oqBcqGGOaakcqWGRbWAcqGGSaalcqWGTbqBcqGGPaqkcqGH9aqpdaWcaaqaamaaqababaGaemOqai0aaSbaaSqaaiabdUgaRbqabaGccqGGOaakcqWGPbqAcqGGSaalcqWGTbqBcqGGPaqkaSqaaiabdMgaPbqab0GaeyyeIuoaaOqaamaaemaabaGaem4uamfacaGLhWUaayjcSdGaeyOeI0IaemyBa0Maey4kaSIaeGymaedaaaaa@4880@

and

σ2(k,m)=∑i(Bk(i,m)−μ(k,m))2|S|−m+1
 MathType@MTEF@5@5@+=feaafiart1ev1aaatCvAUfKttLearuWrP9MDH5MBPbIqV92AaeXatLxBI9gBaebbnrfifHhDYfgasaacH8akY=wiFfYdH8Gipec8Eeeu0xXdbba9frFj0=OqFfea0dXdd9vqai=hGuQ8kuc9pgc9s8qqaq=dirpe0xb9q8qiLsFr0=vr0=vr0dc8meaabaqaciaacaGaaeqabaqabeGadaaakeaaiiGacqWFdpWCdaahaaWcbeqaaiabikdaYaaakiabcIcaOiabdUgaRjabcYcaSiabd2gaTjabcMcaPiabg2da9maalaaabaWaaabeaeaacqGGOaakcqWGcbGqdaWgaaWcbaGaem4AaSgabeaakiabcIcaOiabdMgaPjabcYcaSiabd2gaTjabcMcaPiabgkHiTiab=X7aTjabcIcaOiabdUgaRjabcYcaSiabd2gaTjabcMcaPiabcMcaPmaaCaaaleqabaGaeGOmaidaaaqaaiabdMgaPbqab0GaeyyeIuoaaOqaamaaemaabaGaem4uamfacaGLhWUaayjcSdGaeyOeI0IaemyBa0Maey4kaSIaeGymaedaaaaa@546E@

Be the mean and the variance of the scores obtained by *m*-mers of the reference sequence when matched against sequence *H*_*k*_. The term |*S*| indicates the length of the reference sequence. The score of each *m*-mer is standardized into a χ^2 ^relative score:

χk2(i,m)=(Bk(i,m)−μ(k,m))2σ2(k,m)
 MathType@MTEF@5@5@+=feaafiart1ev1aaatCvAUfKttLearuWrP9MDH5MBPbIqV92AaeXatLxBI9gBaebbnrfifHhDYfgasaacH8akY=wiFfYdH8Gipec8Eeeu0xXdbba9frFj0=OqFfea0dXdd9vqai=hGuQ8kuc9pgc9s8qqaq=dirpe0xb9q8qiLsFr0=vr0=vr0dc8meaabaqaciaacaGaaeqabaqabeGadaaakeaaiiGacqWFhpWydaqhaaWcbaGaem4AaSgabaGaeGOmaidaaOGaeiikaGIaemyAaKMaeiilaWIaemyBa0MaeiykaKIaeyypa0ZaaSaaaeaacqGGOaakcqWGcbGqdaWgaaWcbaGaem4AaSgabeaakiabcIcaOiabdMgaPjabcYcaSiabd2gaTjabcMcaPiabgkHiTiab=X7aTjabcIcaOiabdUgaRjabcYcaSiabd2gaTjabcMcaPiabcMcaPmaaCaaaleqabaGaeGOmaidaaaGcbaGae83Wdm3aaWbaaSqabeaacqaIYaGmaaGccqGGOaakcqWGRbWAcqGGSaalcqWGTbqBcqGGPaqkaaaaaa@5252@

The overall relative score for the *m*-mer at position *i *of the reference sequence is finally defined as the sum of the relative score contributions of each homologous sequence:

χ2(i,m)=∑kχk2(i,m)
 MathType@MTEF@5@5@+=feaafiart1ev1aaatCvAUfKttLearuWrP9MDH5MBPbIqV92AaeXatLxBI9gBaebbnrfifHhDYfgasaacH8akY=wiFfYdH8Gipec8Eeeu0xXdbba9frFj0=OqFfea0dXdd9vqai=hGuQ8kuc9pgc9s8qqaq=dirpe0xb9q8qiLsFr0=vr0=vr0dc8meaabaqaciaacaGaaeqabaqabeGadaaakeaaiiGacqWFhpWydaahaaWcbeqaaiabikdaYaaakiabcIcaOiabdMgaPjabcYcaSiabd2gaTjabcMcaPiabg2da9maaqafabaGae83Xdm2aa0baaSqaaiabdUgaRbqaaiabikdaYaaakiabcIcaOiabdMgaPjabcYcaSiabd2gaTjabcMcaPaWcbaGaem4AaSgabeqdcqGHris5aaaa@42F5@

χ^2 ^scores are computed only when *B*_*k *_*(i, m) *> μ *(k,m) *for each homologous sequence *H*_*k*_, otherwise it is set to zero. Concerning suitable values for the motif length *m *that has to be considered, in the experiments we present in this article we ran the algorithm on size *m *= 8 and *m *= 12, computing for each mean and variance values: longer motifs or regions are detected by combining and merging shorter ones, as explained in the following section.

#### Merging motifs

Very often, the regulation of the transcription of an eukaryotic gene is the result of the simultaneous action of different TFs. Binding sites for cooperative or competitive factors are often adjacent or overlapping each other, with the result that regions longer than a single site are often found to be conserved throughout different species. In order to detect explicitly these regions, in the second step the algorithm merges motifs adjacent in the reference sequence (e.g., motif *m*_1 _in position *i *of length *l *and motif *m*_2 _in position *i + l*), if their best occurrences (the ones that were used to compute their scores) are adjacent in all the homologs.

Since the occurrences of the two motifs do not overlap and are independent, the sum of the original *B*_*i *_scores of two motifs *m*_1 _and *m*_2 _(denoted here for sake of simplicity as *B*_*i*_*(m*_1_*) *and *B*_*i*_*(m*_2_*)*) has mean *μ*_1 *i *_+ *μ*_2 *i *_and variance σ^2^_1 *i*_+σ^2^_2 *i*_, that is, are the sum of mean and variance of the first and second motif *B*_*i *_scores, respectively, computed according to the motifs' length for each homologous sequence *H*_*i*_. The resulting χ^2 ^score of the merged motif can be then defined as:

χ2(m1m2)=∑i(Bi(m1)+Bi(m2)−μ1i−μ2i)2σ1i2+σ2i2
 MathType@MTEF@5@5@+=feaafiart1ev1aaatCvAUfKttLearuWrP9MDH5MBPbIqV92AaeXatLxBI9gBaebbnrfifHhDYfgasaacH8akY=wiFfYdH8Gipec8Eeeu0xXdbba9frFj0=OqFfea0dXdd9vqai=hGuQ8kuc9pgc9s8qqaq=dirpe0xb9q8qiLsFr0=vr0=vr0dc8meaabaqaciaacaGaaeqabaqabeGadaaakeaaiiGacqWFhpWydaahaaWcbeqaaiabikdaYaaakiabcIcaOiabd2gaTnaaBaaaleaacqaIXaqmaeqaaOGaemyBa02aaSbaaSqaaiabikdaYaqabaGccqGGPaqkcqGH9aqpdaaeqbqaamaalaaabaGaeiikaGIaemOqai0aaSbaaSqaaiabdMgaPbqabaGccqGGOaakcqWGTbqBdaWgaaWcbaGaeGymaedabeaakiabcMcaPiabgUcaRiabdkeacnaaBaaaleaacqWGPbqAaeqaaOGaeiikaGIaemyBa02aaSbaaSqaaiabikdaYaqabaGccqGGPaqkcqGHsislcqWF8oqBdaWgaaWcbaGaeGymaeJaemyAaKgabeaakiabgkHiTiab=X7aTnaaBaaaleaacqaIYaGmcqWGPbqAaeqaaOGaeiykaKYaaWbaaSqabeaacqaIYaGmaaaakeaacqWFdpWCdaqhaaWcbaGaeGymaeJaemyAaKgabaGaeGOmaidaaOGaey4kaSIae83Wdm3aa0baaSqaaiabikdaYiabdMgaPbqaaiabikdaYaaaaaaabaGaemyAaKgabeqdcqGHris5aaaa@61D1@

Merging is performed by the algorithm only on motifs that in the first step had positive χ^2 ^score, and thus were more conserved than the average in each homologous sequence. This step is iterated for each position of the reference sequence, that is, single motifs are first compared to the adjacent ones; then, motifs resulting from merging are compared to the adjacent ones, and so on until no further merging is possible. The result is that in this way long conserved regions can be detected, but the regions have to be conserved also *locally*: they must be built by fragments that, taken singularly, fit our model for conserved TFBSs. Thus, in the merging step the algorithm is able to detect regions of size 16, 20, 24, and so on.

#### Input parameters

The only parameters needed by the algorithm, other than the species of origin of the input sequence, are the motifs' size and the maximum number of substitutions allowed when collecting occurrences. As introduced before, in our experiments we used oligos of length *m *= 8 and 12, with *e *= 2 and 3 substitutions, respectively. The choice of these parameters was based first of all on the parameters used in the original Weeder algorithm, and also on studies that showed how variation of 25% of the sequence seems to be a critical value, at least for human-rodent comparisons. This, in turn, implies that also longer regions must present at most 25% of substitutions in their occurrences in the homologs. Usually, TFBSs presenting 30% or more mutations in their homologs are in fact much less likely to preserve their function [37].

#### Output

In a typical application, like a human-rodents comparison, several overlapping motifs (before and after merging) with positive χ^2 ^scores are found. To trim down the size of the output, the algorithm avoids reporting motifs that overlap by more than 2 bps for eightmers, 3 bps for twelve-mers, and 4 bps for longer motifs, with a simple top-down greedy procedure. If a motif overlaps another motif with higher score by more than the defined number of nucleotides, it is removed from the ranked output of motifs.

**Figure 1:**
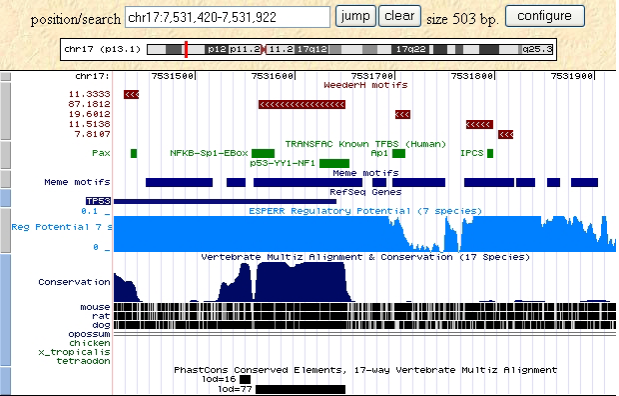
**An example of the output of WeederH**. An example of the output of WeederH, showing the highest scoring motifs in core promoter and first non-coding exon of the human p53 gene, compared to mouse and rat homologs. Motifs are displayed within the UCSC genome browser. Sites annotated in the TRANSFAC database are shown in green. The longest (and highest scoring) motif encompasses several adjacent sites. Regulatory potential (RP) score [27] and phastCons [45] tracks are also displayed (see Results), together with the motifs output by MEME [38] on the same dataset (run in "oops" mode).

An example of a typical output of the algorithm is shown in Figure 1, for the 500 bp upstream and non-coding first exon of the p53 gene of human, mouse, and rat. The highest scoring motifs are shown, displayed within a UCSC genome browser window. Known TFBSs annotated for the human gene in the TRANSFAC database are also shown. It can be seen how a quite long region has been reported, on which adjacent binding sites map, while other shorter motifs are scattered along the sequence, the distal ones falling within a region not deemed to be conserved according to genomic alignments ("Conservation" track). We also show the location on the human sequence of motifs predicted by MEME [38] (run in one occurrence per sequence mode), that cover most of the sequence itself: in fact, since algorithms of this kind are mainly aimed at the detection of very subtle similarities [2], the high level of overall conservation and the few sequences available lead to the prediction of several long significantly conserved motifs.

### Experimental setup

To test the algorithm, we used data taken from the ABS database [39], a collection of experimentally validated transcription factor binding sites conserved in at least two species, together with the homologous promoters containing them. We retrieved from the database 99 sets of homologous sequences 500 bps long, containing a total of 302 experimentally validated binding sites. Seven sequence sets contained human-mouse-rat sequences, 66 a human-mouse pair, 17 a human-rat pair, and 9 mouse- rat sequences. We used these data first to build simulated datasets, then to test the algorithm on real orthologous sequences.

### Results on simulated sequences

We built a dataset of simulated sequences as follows. For each human sequence retrieved from the database, we generated simulated mouse and rat sequences by using the Dawg program [40], that permits the simulation of sequence evolution also including insertion and deletions. We set different substitution rates yielding different sequence identity percentages, while gap and the other parameters needed were estimated by the Dawg algorithm from the alignment of the sequences retrieved from the database. Since our algorithm selects motifs according to maximum substitution rates, rather than defining substitution rates also for the evolution of binding sites we chose to use the original sites annotated in the ABS database. Thus, once a simulated rodent sequence set was generated, we selected a single site annotated in the human sequence and we planted its annotated homologs in the rodent sequences, together with the five nucleotides flanking them on each side. Motifs were planted at their original positions, since WeederH scores motifs according to their conservation in position. In this way, we obtained 254 sequence sets, each containing a single planted motif. Eighteen sets were composed by human-mouse-rat sequences, 185 by a human-mouse pair, and 51 contained human-rat sequences.

We assessed the performance of the algorithm by using different measures. First of all, at the nucleotide level, we calculated the percentage of the nucleotides of the true sites planted in the sequences that were predicted as part of a conserved motif by the algorithm (nucleotide coverage – Nc). This measure is equivalent to sensitivity (ratio of true positives vs. overall sites length). Then, at the site level, by defining a site as correctly predicted if either at least eight of its nucleotides or at least 75% of the site nucleotides overlapped a predicted motif (site coverage – Sc). In order to have an estimate of the false positive predictions of the algorithm we also computed the overall length of the motifs predicted by the algorithm in each sequence set, that is, the percentage of the reference sequence covered by motifs (%pred), and according to this the specificity (ratio of true negatives vs. overall length of the part of the reference sequence not containing a site).

Table 1 shows the results of the algorithm applied to simulated sequences with an average identity of 50%, which has been shown to be average identity percentage on 2,000 bp regions in non-coding human-rodent homologous sequences [5,41]. The performance varies according to the χ^2 ^score threshold used. At threshold value 2 we obtain 85% and 93% of nucleotide and site coverage, respectively, but 44% of the reference sequence is covered by motifs, with a specificity of .54. Increasing the score threshold significantly lowers the number of motifs reported, with the percentage of motifs correctly predicted remains at satisfactory values. With threshold 7.5, the algorithm identifies more than 85% of the planted sites, with only 15% of the reference sequence covered by motifs (specificity .85).

**Table 1:** Performance of WeederH on simulated promoter sets

**χ^**2 **^Score Threshold**	**%pred**	**Nc**	**Sc**	**Sp**
**0**	82.98	94.99	99.21	0.12
**1**	57.97	88.51	96.45	0.39
**2**	44.50	85.74	93.30	0.54
**3**	34.98	82.89	91.73	0.64
**5**	24.00	78.87	89.37	0.76
**7.5**	15.59	74.23	85.82	0.85
**10**	10.90	67.44	77.95	0.90
**12**	8.33	64.30	73.62	0.93
**15**	6.15	60.23	69.29	0.95
				
**FP**	10.54	61.48	72.44	0.90

Performance of WeederH at different χ^2 ^score thresholds on the 254 simulated sequence sets. %pred indicates the percentage of nucleotides of the reference sequence belonging to a motif output by the algorithm. Nc indicates the nucleotide coverage, the percentage of nucleotides in annotated sites matched by a predicted motif (equalling sensitivity); Sc indicates the site coverage, the percentage of annotated sites matched (at least by 75% of their length) by a motif predicted by WeederH; finally, Sp is specificity. The bottom row reports the performance of the Footprinter algorithm on the same set.

To make a comparison, the core of the Footprinter algorithm is based on the same idea, finding matching oligos in the sequences examined, and computing a parsimony score according to the number of mismatches in motifs' instances and the phylogenetic relationship among the species investigated. When no substitution was allowed in motifs instances, Footprinter yielded a nucleotide coverage (sensitivity) of 61%, and a site coverage of 72%, with motifs predicted only on small fraction of the reference sequence (10% – specificity .9). With threshold 10, however, WeederH reached the same specificity, but with sensitivity of .67 and site coverage of about 78%, respectively.

**Figure 2:**
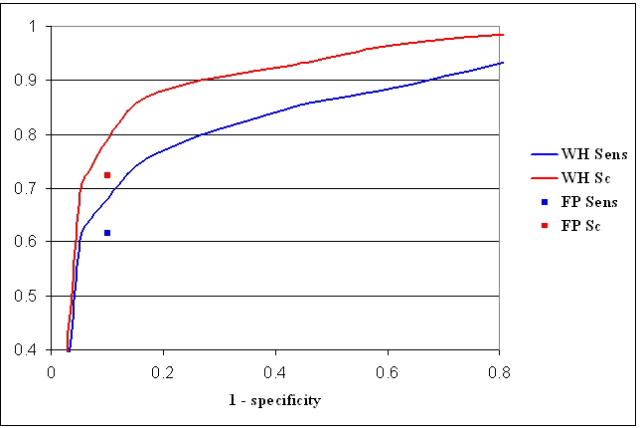
**Performance of WeederH on the simulated promoter set**. Performance of WeederH at different χ^2 ^score thresholds on 254 simulated promoter sequence sets. The plot shows the ROC curve (sensitivity vs. 1-specificity), blue line, and the site coverage Sc versus 1-specificity, red line. Blue and red boxes indicate the performance of Footprinter (sensitivity and Sc).

Other than yielding lower accuracy, the parsimony score employed by Footprinter makes a fine grained ranking of motifs more difficult, especially when few (two or three) homologous sequences are examined. Allowing one or two substitutions when searching for conserved motifs (eightmers with two substitutions are exactly the same parameters employed by WeederH) improved the results up to 99% when allowing two substitutions, but also increased significantly the percentage of the reference sequence covered by a motif: 99% with two substitutions (as in WeederH with no score threshold employed, data not shown) and 72% with one substitution. Figure 2 shows the ROC curve obtained by WeederH at different χ^2 ^values, plotting sensitivity and site coverage vs. (1-specificity).

### Results on homologous human-rodent promoters

**Figure 3:**
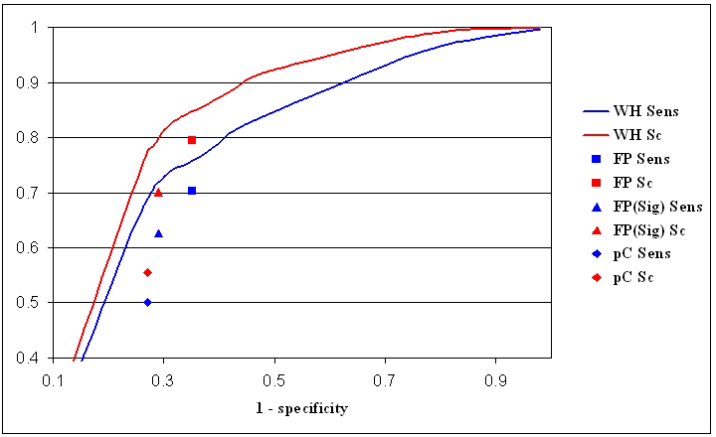
**Performance of WeederH on the ABS promoter set**. Performance of WeederH at different χ^2 ^score thresholds on the 99 promoter sequence sets retrieved from the ABS database. The plot shows the ROC curve (sensitivity vs. 1-specificity), blue line, and the site coverage Sc versus 1-specificity, red line. Blue and red boxes indicate the performance of Footprinter (sensitivity and Sc), blue and red triangles Footprinter with significance measure, blue and red diamond the results of phastCons most conserved regions.

As a further test, we applied the algorithm to the original datasets retrieved from the ABS database (sequences are available as Additional file 1, and the location of the conserved TFBSs as Additional file 2), composed of 90 sets of human-rodent homologous promoters and 9 mouse-rat sequence sets. The results, at different χ^2 ^values are summarized in Table 2 and Figure 3 (see Additional file 3 for the full WeederH output, and Additional file 4 for a more detailed performance analysis).

**Table 2:** Performance of WeederH on the ABS promoter set

**χ^**2 **^Score Threshold**	**%pred**	**Nc**	**Sc**	**Sp**
**None**	98.37	99.70	100.0	0.02
**0**	78.90	95.77	98.68	0.23
**1**	51.04	83.91	91.72	0.52
**1.5**	45.79	81.42	89.07	0.57
**2**	41.93	78.26	86.75	0.61
**2.5**	39.53	76.35	85.10	0.64
**3**	37.55	75.24	84.11	0.66
**4**	34.55	73.96	82.45	0.69
**4.5**	33.26	72.42	80.79	0.70
**5**	32.30	71.56	79.14	0.71
**5.5**	31.44	70.77	78.48	0.72
**6**	30.58	68.96	77.48	0.73
**8**	27.51	64.81	72.18	0.75
				
**FP**	37.18	70.32	79.47	0.65
**FPSIG**	31.55	62.61	70.19	0.71
				
**phastCons**	25.97	50.02	55.51	0.73

Performance of WeederH at different χ^2 ^score thresholds on the 99 orthologous promoter sets retrieved from the ABS database. %pred indicates the percentage of nucleotides of the reference sequence belonging to a conserved motif output by the algorithm. Nc indicates the nucleotide coverage, the percentage of nucleotides in annotated sites matched by a predicted motif; Sc indicates the site coverage, the number of annotated sites matched (at least by 75% of their length) by a motif predicted by WeederH; Sp is specificity. The next two rows report the performance of the Footprinter algorithm on the same set, using parsimony score alone (FP), and with the introduction of significance estimation (FPSIG). Finally, the bottom row shows the same data for the phastCons most conserved tracks available at the UCSC genome browser (on genomes: human and mouse, March 2006; rat, November 2004).

The first piece of information that can be gathered is that virtually all the reference sequence is covered by a motif satisfying the substitution threshold, as shown by the first row of the table that reports the motifs found regardless of the χ^2 ^score threshold. But, the annotated sites in the sequences have indeed a positive χ^2 ^score, since using a threshold of 0 yields coverage of about 99% at the site level and 96% at the nucleotide level. In this case, however, the motifs output still cover about 79% of the reference sequence. Increasing the χ^2 ^score threshold to values ≥ 2 has the effect of lowering the fraction of the reference sequence covered by a conserved motif to 30–40%, while the site coverage falls less sharply, with about 80–85% of the annotated sites correctly predicted. It should be noticed that in this case higher performances are obtained at lower threshold values, nevertheless with lower specificity values. This is due to the fact that the sequence sets analyzed are in general more conserved than the artificial sets we generated (around 60–65% of identity), and thus motifs stand out less well with respect to the rest of the sequences. A single sequence can also contain more than one annotated site. Moreover, rather than be spread randomly along the sequences as in the previous case, now it is more likely to find conserved blocks within the sequences, thus yielding a higher number of oligos satisfying the substitution thresholds employed by the algorithm.

The number of sites correctly predicted by the algorithm is anyway again quite satisfactory, but in this case assessing how many false positives are reported is far from being straightforward. First of all, one should have an estimate of how many functional sites should be expected inside the 500 bp promoter of a mammalian gene. Then, according to the species included in the analysis, how many sites should be expected to be conserved, a number that for human-mouse comparisons has been estimated to be ranging from 60 to 72% [37,42-44]. In other words, the region analyzed might also contain other functional sites not conserved in the other species. Finally, the conservation of functional sites often spans a region longer than the sites themselves, increasing the number of predicted nucleotides.

We then ran Footprinter also on this sequence set. Again, the best performance (79% at the site level, 70% at the nucleotide level) was obtained by using a parsimony score threshold of 0 (no substitution allowed in motifs' occurrences), with motifs covering more than 37% of the reference sequence. With χ^2 ^score threshold of 3.0 WeederH motifs covered the same percentage of the reference sequence, with 84% of success at the site level and 76% at the nucleotide level (see Table 1). Thus, with the same specificity, WeederH yields a higher sensitivity. Introducing for Footprinter significance evaluation of motifs (using the different significance settings available and trying different combinations) did not improve the results, increasing the specificity but also reducing the percentage of correctly identified sites to 70%. The same specificity was reached by WeederH with χ^2 ^score threshold of 5.0, but with 79% of the annotated sites correctly predicted.

**Figure 4:**
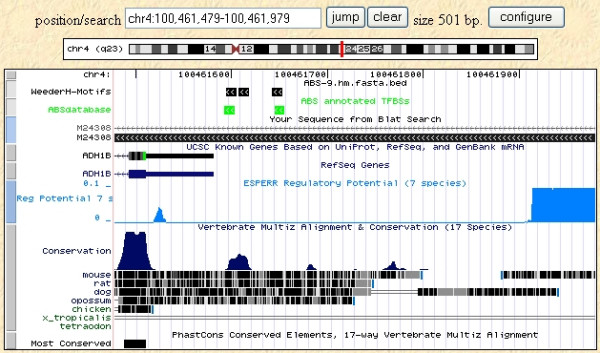
**WeederH output on the 500 bp promoter of the ADH1B gene (as defined in the ABS database)**. Top three motifs output by WeederH (top track), ABS annotated sites (in green), sequence of the ABS database (obtained by BLAT search) and predictions according to RP score [27], light blue) and phastCons ([45], bottom track). Notice how neither of the latter two methods reports anything significantly conserved in the core promoter itself, where motifs are located.

**Figure 5:**
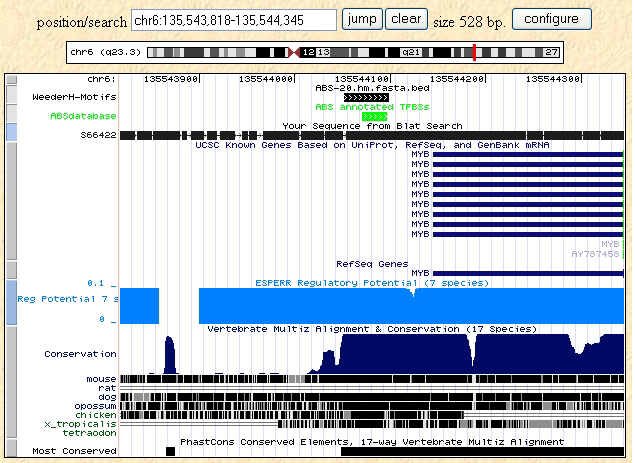
**WeederH output on the promoter and 5'UTR of the MYB gene (as defined in the ABS database)**. Highest scoring motif output by WeederH (top track), ABS annotated site (in green), sequence of the ABS database (S66422 – aligned with BLAT), and conserved regions predicted by RP score [27], light blue) and phastCons ([45], bottom track). Notice how both the latter methods predict conserved long regions spanning most of the promoter itself, making difficult the identification of single conserved TFBSs.

We also compared our results to the phastCons annotations [28,45] available at the UCSC genome browser [13]. The overall percentage of the reference sequences used in this test annotated as "most conserved" is around 25%, and about 55% of the sites annotated in the database are covered (50% at the nucleotide level). Although the tracks are generated by comparing human to all the vertebrate genomic sequences available, instead of rodents alone (and vice versa), this result nevertheless highlights the fact that methods like phastCons are better suited to identify large conserved regions rather than single sites. Examples are shown in Figures 4 and 5, with the results of WeederH compared to the conserved regions predicted by phastCons and RP [27] available at the UCSC genome browser. Indeed, these examples show typical situations in which the two methods either do not detect any conserved element in the core promoter because single TFBSs are too small to reach a significant level of conservation (Figure 4), or can single out only large conserved regions (Figure 5, as also in the p53 example, see Figure 1). This is also a drawback deriving from the usage of a single, global threshold of significance. These examples show how WeederH can work at a much more fine grained level of detail, actually being able to identify correctly conserved TFBSs either with little or, vice versa, a very high level of overall sequence conservation.

**Figure 6:**
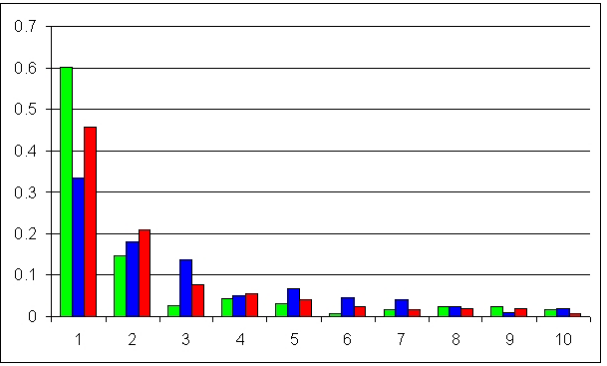
**WeederH ranking of the sites annotated in the simulated and ABS promoter sets**. Ranking of the motifs output by WeederH matching a planted site in the simulated promoter set (green bars) and the ABS promoter set (red and blue bars, see text for explanation).

Given the difference in performance on the same threshold values for the artificial and real datasets, it might seem that the problem of defining a significance threshold remains, only recast at a relative level, that is, depending on the overall degree of conservation of the sequences investigated. However, useful information can be gathered by examining the ranking, in the lists output by the algorithm, of the motifs matching a planted site. The overall distribution is shown in Figure 6. In artificial sequences, more than 60% of the planted sites matched a top-scoring motif, and nearly 80% one of the first three. Also in the real promoter case, motifs corresponding to a functional site tend to appear within the first five positions (blue bars in the histogram of Figure 6), thus providing further evidence to the fact that indeed the measure used by the algorithm highlights conserved real TFBSs, that are as we stated in the introduction, "more conserved than the rest". One third of the 302 sites are matched by the highest ranking motif, 17% by a second-ranking motif and 13% by the third one. 75% of the annotated sites are matched by a motif ranking among the first five, 90% among the first ten. The percentage is increased if we remove from the ranking those higher-scoring motifs that match an annotated site, in other words, if we count in the ranking of a motif only those preceding it that do not match an annotated site (hence "putative false positives"). In this case, almost half of the sites are matched by the highest ranking motif, or by a motif that is preceded by other motifs matching a solution (red bars in Figure 6). Vice versa, in 62 sequence sets out of 99 the highest scoring motif matched an annotated site (or more than one, since the algorithm can report regions longer than a single site, that hence can contain more than one site), and in 91 out of 99 one of the first three motifs matched an annotated site. A rapid inspection of the highest scoring motifs not matching a site annotated in the ABS database, however, revealed that in all the cases but one the highest scoring motif matched at least one site annotated in TRANSFAC, or a signal like TATA- or CAAT-boxes that given their constrained position are perfect candidates to be picked by the algorithm.

The scoring function employed by the algorithm seems thus to be reliable, and scanning the output list from top to bottom is very likely to produce satisfactory results. An interesting result is also the fact that in the case of mouse-rat comparisons, where the sequences presents a much higher degree of similarity, the percentage of annotated sites correctly identified remains fixed at 90% even at high score thresholds, showing that true sites are higher scoring also in the presence of a higher level of sequence conservation.

### Finding conserved distal motifs and regions

As we have shown in the previous section, the scoring function employed by the algorithm can successfully discriminate motifs and regions corresponding to true functional sites. The dataset we used for the test, however, was composed of sequences carefully selected, in other words, truly orthologous promoters. In this way, the positional conservation term in the scoring function quite naturally yields the best results. Very often, however, the selection of the input sequences is far from being straightforward: even in largely annotated genomes like human and mouse, genes present several different transcription start sites, alternative promoters, and so on, making difficult the choice of which "upstream" region has to be considered. Moreover, in other recently sequenced genomes like dog, no transcripts are often available, and genes are annotated only starting from the ATG codon [32]. To assess whether the scoring scheme employed is efficient also in the case of longer sequences less carefully selected we performed further tests.

**Figure 7:**
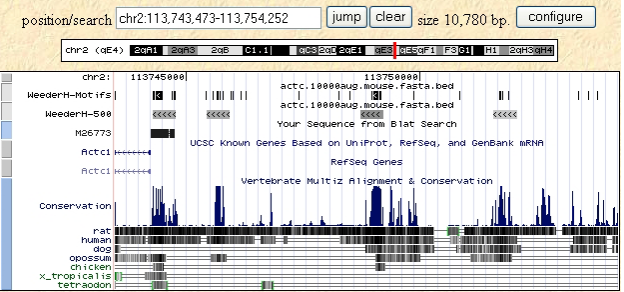
**WeederH identifies the promoter and the three annotated enhancers upstream of the mouse actin, alpha cardiac gene**. Highest scoring motifs predicted by WeederH in the 10,000 bps region upstream of the ATG codon of the mouse actin alpha cardiac gene, displayed within the UCSC genome browser. Track "Weeder H motifs" shows the location of the motifs; the track "Weeder-H" 500 shows 500 bps regions in which the average 12-mer χ^2 ^score is greater than 1. Track "Your sequence from Blat Search" shows the location of the original promoter retrieved from the ABS database. The three regions, other than the just upstream of the TSS (the promoter), match three experimentally known enhancers of the gene.

The Actin cardiac alpha chain gene (*ACTC*) has the ATG codon located within the second exon, with a fully non-coding first exon. WeederH successfully identified all the 7 sites contained in the 500 bp promoter region retrieved from the ABS database (ABS 3 in Additional file 1). We repeated the experiment, but this time retrieving the 10,000 base pairs upstream of the ATG codon of the mouse and human genes. The results are shown in Figure 7, displayed within a UCSC genome browser window. The topmost track (WeederH motifs) shows the location of the highest scoring motifs. It can be seen that they are clustered around the TSS of the gene, falling within the 500 bp promoter of the ABS database (indicated by the "Your sequence from BLAT search" track). Motifs shown in this area cover all the ABS annotated sites. Also interestingly enough, other clusters of motifs are visible, namely at around -2000, -6000, and -8000 from the TSS. As a matter of fact, three distal enhancers are annotated for the *ACTC *gene, driving developmental and cardiac-muscle specific expression of the gene [46].

To ease the identification of clusters of conserved motifs, we added to the basic algorithm the computation of the average motif χ^2 ^score (for 12-mers) in regions 500 bp long. Figure 7 shows the regions with average 12-mer χ^2 ^score greater than 1, matching the experimentally annotated enhancers.

**Figure 8:**
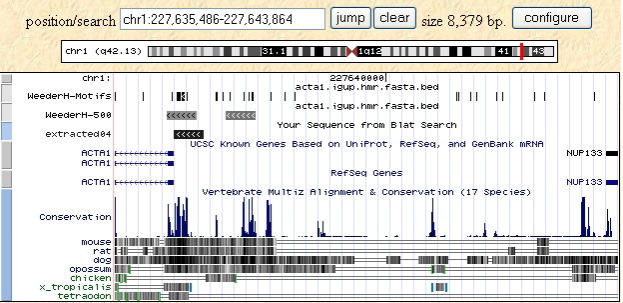
**WeederH identifies the promoter and the annotated enhancer upstream of the human skeletal actin gene**. Highest scoring motifs predicted by WeederH in the intergenic region upstream of the ATG codon of the human skeletal actin gene, displayed within the UCSC genome browser. Track "Weeder H motifs" shows the location of the motifs; the track "Weeder-H" 500 shows 500 bps regions in which the average 12-mer χ^2 ^score is higher than 1. The two regions selected, are the promoter and an annotated enhancer located at about 1500 bps upstream of the TSS [47]. Track "Your sequence from Blat Search" shows the location of the original promoter retrieved from the ABS database.

Another example is the Actin, skeletal muscle gene (*ACTA1*, ABS 4 in Additional file 1). In this case, we retrieved for human, mouse, and rat the whole intergenic region (of about 7000 bps) upstream of the gene. In this case, two regions were selected as densely populated of significant motifs (see Figure 8): the core promoter, again, and another region at around -1,500 matching an experimentally validated enhancer [47].

These examples, as well as other tests we performed, show how the conservation principle the algorithm is based on can work also on input sequences whose size spans well outside the typical length of a core promoter, where positional conservation is looser. While not explicitly devised for the identification of distal enhancers, WeederH nevertheless can be applied to cases where the exact location of the TSS of a gene in different species is not available, or in general to identification of conserved motifs located at several hundreds of base pairs of distance from the reference point selected, on which other methods that do not compute global sequence alignments cannot be applied.

## Conclusion

The ever increasing availability of annotated genomic sequences, as well as the observation that several non coding regulatory sequence elements are highly conserved throughout different species, have made phylogenetic footprinting one of the most widely used approaches to the identification of sequence cis-acting elements regulating gene expression. The algorithm we presented in this work was aimed at overcoming some of the drawbacks of the methods currently used, namely, the need of reliable genomic alignments and/or descriptors for the binding specificity of transcription factors. The introduction of a relative scoring strategy, moreover, bypasses the problem of defining global significance thresholds. The algorithm is also quite efficient, requiring less than one minute for a typical promoter analysis and a few minutes for sequences a few kbp long. The results we obtained from the tests we performed show that the algorithm can reliably predict conserved TFBSs in homologous promoters, with better performance over existing methods and annotations, but also can identify conserved sites and regions in longer sequences. Clearly, other methods are more suited for the discovery of distal regions and enhancers, that can be located at several thousands or millions of base pairs from the gene they regulate; nevertheless, WeederH can provide significantly more information than traditional motif-finding algorithms.

## Methods

### Computing motifs expected frequency values

The scoring function employed by WeederH is based on the comparison of the observed oligo frequencies with expected values. The term *E(s,d,k)*, indicating the expected frequency of a given oligo s with *d *substitutions in the species of origin of sequence *H*_*k *_is computing according to the observed frequency of oligos within d substitutions from s in intergenic regions of the species sequence *H*_*k *_is taken from:

E(s,d,k)=∑s′∈N(s,d)E(s′,k)
 MathType@MTEF@5@5@+=feaafiart1ev1aaatCvAUfKttLearuWrP9MDH5MBPbIqV92AaeXatLxBI9gBaebbnrfifHhDYfgasaacH8akY=wiFfYdH8Gipec8Eeeu0xXdbba9frFj0=OqFfea0dXdd9vqai=hGuQ8kuc9pgc9s8qqaq=dirpe0xb9q8qiLsFr0=vr0=vr0dc8meaabaqaciaacaGaaeqabaqabeGadaaakeaacqWGfbqrcqGGOaakcqWGZbWCcqGGSaalcqWGKbazcqGGSaalcqWGRbWAcqGGPaqkcqGH9aqpdaaeqbqaaiabdweafjabcIcaOiqbdohaZzaafaGaeiilaWIaem4AaSMaeiykaKcaleaacuWGZbWCgaqbaiabgIGiolabd6eaojabcIcaOiabdohaZjabcYcaSiabdsgaKjabcMcaPaqab0GaeyyeIuoaaaa@486E@

where *N(s,d) *is the set of oligos differing from s in no more than *d *positions. Frequency values for eightmers (*E(s,k)*) were retrieved from the RSAT Tools database [48], while the expected frequency of longer oligos is computed starting from the eightmer frequencies with a seventh-order Markov model.

For oligos longer than 8 nts, we modeled the expected frequency by using a seventh order Markov chain. In other words, let *p = p*_1 _.... *p*_*n *_be an *n*-mer, with *n *greater than 8:

Exp(p)=Exp(p1...p8)∏i=9nP(pi|pi−7...pi−1)
 MathType@MTEF@5@5@+=feaafiart1ev1aaatCvAUfKttLearuWrP9MDH5MBPbIqV92AaeXatLxBI9gBaebbnrfifHhDYfgasaacH8akY=wiFfYdH8Gipec8Eeeu0xXdbba9frFj0=OqFfea0dXdd9vqai=hGuQ8kuc9pgc9s8qqaq=dirpe0xb9q8qiLsFr0=vr0=vr0dc8meaabaqaciaacaGaaeqabaqabeGadaaakeaacqWGfbqrcqWG4baEcqWGWbaCcqGGOaakcqWGWbaCcqGGPaqkcqGH9aqpcqWGfbqrcqWG4baEcqWGWbaCcqGGOaakcqWGWbaCdaWgaaWcbaGaeGymaedabeaakiabc6caUiabc6caUiabc6caUiabdchaWnaaBaaaleaacqaI4aaoaeqaaOGaeiykaKYaaebCaeaacqWGqbaucqGGOaakcqWGWbaCdaWgaaWcbaGaemyAaKgabeaakiabcYha8jabdchaWnaaBaaaleaacqWGPbqAcqGHsislcqaI3aWnaeqaaOGaeiOla4IaeiOla4IaeiOla4IaemiCaa3aaSbaaSqaaiabdMgaPjabgkHiTiabigdaXaqabaGccqGGPaqkaSqaaiabdMgaPjabg2da9iabiMda5aqaaiabd6gaUbqdcqGHpis1aaaa@5CF3@

where *P *(*p*_*i *_| *p*_*i-7 *_... *p*_*i-1*_) is the conditional probability of having nucleotide *p*_*i *_preceded by nucleotides *p*_*i-7 *_... *p*_*i-1*_, computed by using the expected frequencies of 8-mers:

P(pi|pi−1...pi−7)=Exp(pi−7pi−6...pi−1pi)∑n∈{A,C,G,T}Exp(pi−7pi−6...pi−1n)
 MathType@MTEF@5@5@+=feaafiart1ev1aaatCvAUfKttLearuWrP9MDH5MBPbIqV92AaeXatLxBI9gBaebbnrfifHhDYfgasaacH8akY=wiFfYdH8Gipec8Eeeu0xXdbba9frFj0=OqFfea0dXdd9vqai=hGuQ8kuc9pgc9s8qqaq=dirpe0xb9q8qiLsFr0=vr0=vr0dc8meaabaqaciaacaGaaeqabaqabeGadaaakeaacqWGqbaucqGGOaakcqWGWbaCdaWgaaWcbaGaemyAaKgabeaakiabcYha8jabdchaWnaaBaaaleaacqWGPbqAcqGHsislcqaIXaqmaeqaaOGaeiOla4IaeiOla4IaeiOla4IaemiCaa3aaSbaaSqaaiabdMgaPjabgkHiTiabiEda3aqabaGccqGGPaqkcqGH9aqpdaWcaaqaaiabdweafjabdIha4jabdchaWjabcIcaOiabdchaWnaaBaaaleaacqWGPbqAcqGHsislcqaI3aWnaeqaaOGaemiCaa3aaSbaaSqaaiabdMgaPjabgkHiTiabiAda2aqabaGccqGGUaGlcqGGUaGlcqGGUaGlcqWGWbaCdaWgaaWcbaGaemyAaKMaeyOeI0IaeGymaedabeaakiabdchaWnaaBaaaleaacqWGPbqAaeqaaOGaeiykaKcabaWaaabuaeaacqWGfbqrcqWG4baEcqWGWbaCcqGGOaakcqWGWbaCdaWgaaWcbaGaemyAaKMaeyOeI0IaeG4naCdabeaakiabdchaWnaaBaaaleaacqWGPbqAcqGHsislcqaI2aGnaeqaaOGaeiOla4IaeiOla4IaeiOla4IaemiCaa3aaSbaaSqaaiabdMgaPjabgkHiTiabigdaXaqabaGccqWGUbGBcqGGPaqkaSqaaiabd6gaUjabgIGiolabcUha7jabdgeabjabcYcaSiabdoeadjabcYcaSiabdEeahjabcYcaSiabdsfaujabc2ha9bqab0GaeyyeIuoaaaaaaa@82C6@

The motivation for the choice of a seventh order model is based on the fact that a high order background models (at least third or fourth) have been proven in several experiments to improve significantly the reliability of motif discovery methods (see for example [49-51]). Moreover, we chose to use directly the *n*-mer count for computing the *Exp(p) *values of *n*-mers, up to the maximum length for which each oligo appeared at least once in the regulatory sequences of the organisms we examined (avoiding the introduction of pseudo-counts to compensate for missing oligo counts). Then, we computed the *Exp(p) *values of oligos longer than 8 nucleotides starting from the eightmer count values.

## Availability and requirements

• **Project name**: WeederH

• **Project home page**: Part of the Motif Discovery Tools web server,  or .

• **Operating systems**: web interface that can be accessed from any OS.

• **Programming language**: C/C++, Java (web interface).

• **Restrictions to use by non-academics**: none.

## Abbreviations

TF, transcription factor; TFBS, transcription factor binding site; TSS, transcription start site; bp, base pair.

## Authors' contributions

GiP came up with the core idea of the algorithm, designed it together with GrP, and finally implemented it; FZ tested extensively the algorithm during its development, and implemented parts of the algorithm itself and of the Web interface. All authors read and approved the final manuscript.

## Supplementary Material

Additional file 1 Orthologous sequence sets. Orthologous sequence sets taken from the ABS database used for testing the algorithm. Click here for file

Additional file 2 Motif solutions. Motifs annotated in the ABS database in the test sequences. Click here for file

Additional file 3 Output of WeederH on orthologous sequence sets. Full output of WeederH on the orthologous sequence sets taken from the ABS database used for testing the algorithm. Click here for file

Additional file 4 WeederH performance on ortholgous promoters. detailed results of WeederH applied to the ABS database data set, split in the different sequence sets and sites. Click here for file
